# Interferon alpha-2a treatment for refractory Behcet uveitis in Korean patients

**DOI:** 10.1186/s12886-018-0719-0

**Published:** 2018-02-20

**Authors:** Ji Hwan Lee, Christopher Seungkyu Lee, Sung Chul Lee

**Affiliations:** Department of Ophthalmology, The Institute of Vision Research, Yonsei University College of Medicine, Yonsei-ro 50-1, Seodaemun-gu, Seoul, Republic of Korea

**Keywords:** Behcet syndrome, Interferon-alpha, Therapeutics, Uveitis

## Abstract

**Background:**

To evaluate therapeutic outcomes of interferon alpha-2a (IFNα2a) treatment in patients with Behcet’s disease who were refractory to immunosuppressive agents.

**Methods:**

This retrospective case series reviewed the medical records of 5 patients with refractory Behcet uveitis from January 2011 to February 2017. IFNα2a was administered at a dose of 3 million IU 3 times per week. Clinical response, relapse rate, and change of visual acuity were evaluated.

**Results:**

The mean age of patients was 39.60 ± 9.21 years, and the median treatment duration was 6 months. Four of the 5 patients (80%) presented with responses to IFNα2a without any uveitis attack during the treatment period. The mean number of uveitis attacks/year per patient during the treatment was 0.40 ± 0.89. The mean log of the Minimum Angle of Resolution visual acuity improved from 1.44 ± 0.38 at baseline to 1.02 ± 0.58 at the final follow up.

**Conclusions:**

IFNα2a is an effective therapy for Behcet uveitis refractory to conventional immunosuppressants in Korean patients.

## Background

Behcet’s disease (BD) is a chronic relapsing multisystem vasculitis mainly characterized by recurrent oral ulceration, genital ulceration, ocular lesions, and skin lesions [1]. Ocular involvement is one of the most serious complication of BD, as repeated attacks of uveitis may result in blindness [2].

Corticosteroid treatment is the mainstay in the management of acute uveitic attacks, and immunosuppressive agents such as cyclosporine and azathioprine are usually effective in long-term management [3, 4]. Interferon alpha-2a (IFNα2a) has been reported to be effective and safe in refractory cases, although the optimal regimen has not yet been established [5–13]. In this study, we aimed to evaluate the efficacy of IFNα2a in Korean patients with Behcet uveitis refractory to immunosuppressive agents.

## Methods

### Patients

We retrospectively reviewed the medical records of 5 patients with refractory Behcet uveitis who were treated with IFNα2a from January 2011 to February 2017. Refractory Behcet uveitis was defined as unresponsive or recurrent uveitis despite combination therapy of immunosuppressive agents and corticosteroids. Patients who were followed up for at least 3 months were included in this study. All the patients met the criteria of the International Study Group for Behcet’s disease [14]. This study was approved by the institutional review board of Severance Hospital, Yonsei University College of Medicine (IRB No.4–2017-0436).

### Interferon alpha-2a treatment

IFNα2a (Roferon-A®; Roche; Basel, Switzerland) was administered at a dose of 3 × 10^6^ IU 3 times per week. All previous immunomodulatory agents were stopped the day before the initiation of IFNα2a. During IFNα2a therapy, oral corticosteroid was tapered to a low dose (5–10 mg/d prednisolone equivalent) or discontinued according to a general tapering schedule (to reduce by 5 mg/day every 1–2 weeks if the dose of prednisolone is 20-40 mg/day, to reduce by 2.5 mg/day every 1–2 weeks if the dose is below 20 mg).

### Assessments

All patients underwent a complete ophthalmologic examination, including best-corrected visual acuity (BCVA), slit lamp biomicroscopy, tonometry, and fundoscopy. Ancillary examinations included fluorescein angiography and optical coherence tomography. Examinations were performed weekly for 2 weeks, every 2 weeks for 1 month, and then once every month. Relapse was defined as two step increase in level of inflammation including anterior chamber cells or vitreous haze [15]. The relapse rate was calculated as attacks per year. Response to IFNα2a therapy was defined as maintenance of inactive disease without any relapse during the treatment period. The mean LogMAR BCVA and the mean number of uveitis attacks per year at baseline and final visit were compared using Wilcoxon signed-rank test. Statistical analyses were performed using SPSS version 23.0 (IBM; Chicago, IL, USA) and a *p*-value< 0.05 was considered statistically significant.

## Results

### Patients

Demographic and clinical characteristics of patients are summarized in Table 1. The mean age of patients was 36.60 ± 9.21 years and 5 patients were male in this study. The mean overall follow up period including the treatment period was 58.80 ± 33.48 months. All patients were Korean. Four patients (80%) presented bilateral involvement. Extraocular manifestations of BD included oral aphthous ulcers and skin lesions in all patients (100%), genital ulcer in 1 patient (20%), gastrointestinal involvement in 2 patients (40%), central nervous system involvement in 1 patient (20%), and epididymitis in 1 patient (20%). Prior to IFNα2a therapy, 3 patients received combination therapy of azathioprine, cyclosporine, or methotrexate, and 2 patients were treated with mycophenolate mofetil.

**Table 1:** Demographic and clinical characteristics of patients with refractory Behcet uveitis

	Patient 1	Patient 2	Patient 3	Patient 4	Patient 5
Age-range at onset (years)	20–30	40–50	20–30	30–40	30–40
Duration of IFNα2a treatment (months)	6	5	28	12	2
Overall follow-up period (months)	51	27	34	72	110
Laterality	Bilateral	Unilateral	Bilateral	Bilateral	Bilateral
Anatomic classification of uveitis	Panuveitis	Panuveitis	Panuveitis	Panuveitis	Panuveitis
Extraocular manifestations of BD	Oral ulcer CNS involvement Epididymitis Arthritis Skin lesion (EN)	Oral ulcer Genital ulcer Skin lesion(EN) GI involvement	Oral ulcer Skin lesion (folliculitis) GI involvement	Oral ulcer Skin lesion (EN)	Oral ulcer Skin lesion(EN)
Previous immunosuppressive treatment	Azathioprine 100 mg/d, Methotrexate 17.5 mg weekly	Cyclosporine 200 mg/d Azathioprine 100 mg/d, Methotrexate 17.5 mg weekly	Cyclosporine 200 mg/d, Azathioprine 100 mg/d	Mycophenolate mofetil 2 g/d	Mycophenolate mofetil 2 g/d
Dose of oral corticosteroid (mg/d prednisolone equivalent), preTx→postTx	40 → 10	15 → 0	20 → 0	40 → 5	15 → 0
Relapse rate (number of uveitis attacks/year), preTx→postTx	2.13 → 2	1.64 → 0	4.00 → 0	1.80 → 0	1.22 → 0
Response to IFNα2a	No	Yes	Yes	Yes	Yes
Adverse events	Flu-like Sx Depression	Flu-like Sx	Flu-like Sx	Flu-like Sx	Flu-like Sx

*BD*: Behcet’s disease, *CNS*: central nervous system, *EN*: erythema nodosum, *GI*: gastrointestinal, *IFNα2a*: interferon alpha-2a, *Sx*: symptoms, *Tx*: treatment

### Interferon alpha-2a treatment

**Fig. 1:**
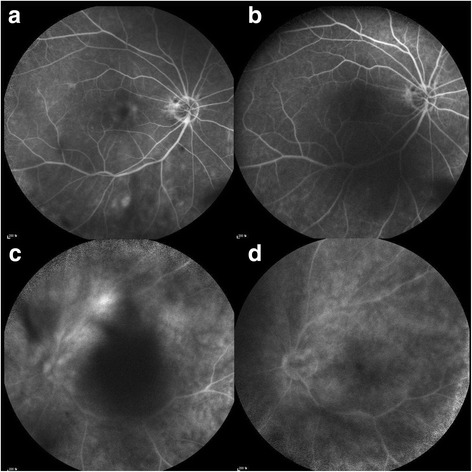
Fluorescein angiographic images of patients with refractory Behcet uveitis. In patient 4, diffuse capillary leakage (**a**) decreased 6 months after interferon alpha-2a (IFNα2a) therapy (**b**). In patient 3, moderate vascluitis at the superior arcade (**c**) was significantly resolved 17 months after the initiation of IFNα2a treatment (**d**)

The median duration of IFNα2a treatment was 6 months (range 2–28 months). Four (80%) of 5 patients showed responses to IFNα2a without any uveitis attack during the treatment period (Fig. 1). The mean number of uveitis attacks per year during the treatment was 0.40 ± 0.89, which decreased from 2.16 ± 1.08 before IFNα2a therapy (*p* = 0.043). Four responsive patients could not discontinue IFNα2a therapy in this study. One patient (20%) received posterior subtenon triamcinolone injection during the treatment period. In 1 unresponsive patient, IFNα2a was switched to infliximab. Visual acuity improved at final visit compared with baseline in all patients. The mean log of the Minimum Angle of Resolution (logMAR) BCVA changed from 1.44 ± 0.38 (Snellen equivalent 20/550) at baseline to 1.02 ± 0.58 (Snellen equivalent 20/209) at final visit (*p* = 0.068). Although the baseline BCVA was 20/200 or less in all patients (100%), the final BCVA of 20/200 or less were observed in 2 patients (40%).

### Adverse events

All patients experienced flu-like symptoms at the beginning of IFNα2a treatment. One patient presented with mild depression, which was relieved by antidepressant medication. No other significant adverse effects were observed during the treatment period.

## Discussion

In this study, we evaluated the efficacy of IFNα2a in patients with Behcet uveitis refractory to immunosuppressive agents. Most patients had good responses to IFNα2a. IFNα2a therapy was maintained in these patients. There were no uveitis attacks during the treatment period in the 4 patients who were responsive to IFNα2a therapy. Visual acuity improved in all patients.

Corticosteroid is the main treatment option for acute attacks of Behcet uveitis. However, its long-term use is limited because of adverse effects. Cyclosporine and azathioprine have been effectively used in Behcet uveitis alone or combined with other immunosuppressants in severe cases [3, 4]. There are, however, some patients who are refractory to immunosuppressive agents, and biological therapies including anti-tumor necrosis factor antibody, anti-interleukin, or interferon can be considered in such cases [16, 17]. Recently, IFNα2a has been reported to be effective for the treatment of refractory Behcet uveitis [5, 7–13, 18, 19].

There is no consensus on the dose and protocol of IFNα2a therapy for Behcet uveitis. In this study, we used a lower-dose regimen of 3 × 10^6^ IU of IFNα2a 3 times per week during the treatment period. The rate of treatment response in our series was 80%, which is similar to that in previous reports using higher doses of IFNα2a [13, 19]. A lower-dose regimen may be associated with fewer treatment-related complications. There were no severe adverse effects in the present study. In contrast, patients with leukopenia or thrombocytopenia have been reported in previous studies using higher doses of IFNα2a [18, 20]. Four responsive patients could not discontinue IFNα2a therapy in this study, which may also have been associated with the lower dose of the regimen. By comparison, 25–50% of patients may discontinue IFNα2a treatment with higher-dose regimens [11, 13].

The relapse rate of uveitis attacks significantly decreased from 2.16 ± 1.08 to 0.40 ± 0.89 after IFNα2a therapy. In the 4 patients who had responses to IFNα2a therapy, there were no uveitis attacks during the treatment period. The efficacy of IFNα2a therapy in terms of uveitis relapse was comparable to recent reports [13, 19]. We confirmed that treatment response without uveitis relapse may be achieved mostly with low-dose continuous IFNα2a therapy in Korean patients.

As refractory Behcet uveitis cases are rare, the major limitations of this study are its retrospective design and the small number of patients. We were, however, able to confirm the efficacy of IFNα2a therapy in a uniform low-dose regimen. Questions regarding the optimal dosage, treatment duration, and treatment protocol of IFNα2a therapy still remain unanswered. A prospective study would be necessary not only to determine the most effective and safest protocol, but also to compare the efficacy of IFNα2a with new biological agents currently under study.

## Conclusions

IFNα2a is an effective therapeutic for Behcet uveitis refractory to other immunosuppressants in Korean patients.

## Abbreviations

BCVA: best-corrected visual acuity
BD: Behcet’s disease
IFNα2a: interferon alpha-2a
LogMAR: log of the minimum angle of resolution
